# Supplementary material for: Identification of photoperiod-induced specific miRNAs in the adrenal glands of Sunite sheep (Ovis aries)
Source: Front Vet Sci. 2022 Jul 22;9:888207. doi: 10.3389/fvets.2022.888207 (PMC9354845; doi:10.3389/fvets.2022.888207)
Supplement: Supplementary file 1 [file Data_Sheet_1.ZIP › Supplementary file/miRDeep2 file.zip!/miRDeep2 file/Novel_Structure/LP42A1/Novel_221.pdf]

Provisional ID	:	NC_019478.2_30782
Score total	:	1.9
Score for star read(s)	:	-1.3
Score for read counts	:	0
Score for mfe	:	1.6
Score for randfold	:	1.6
Score for cons. seed	:	
Total read count	:	493
Mature read count	:	493
Loop read count	:	0
Star read count	:	0

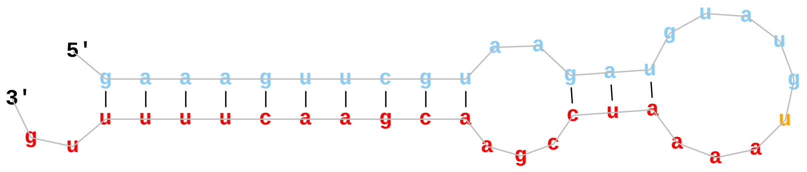

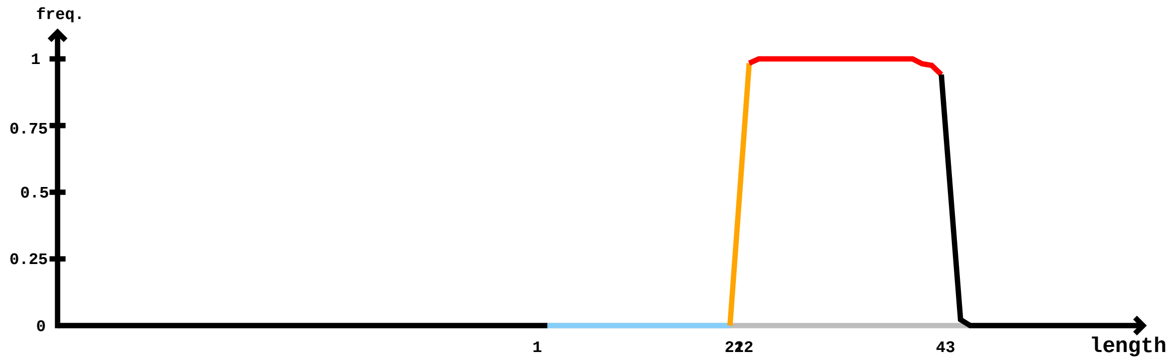

Star Mature

```
5'- gcagaggaaggaaggagggggugucugagaggguauugggcuugggccagaaaguucguaagauguauguaaaaauccgaacgaacuuuuugggcccacccaguauuucaaa   -3'    exp
    .((((.(..............).))))...((((((((((..(((((((((((((((((..(((.........)))...))))))))))).))))))))))))))))....   reads   mm   sample
    ......................................................................aaaaauccgaacgaacuuu.......................   9       0    seq
    ......................................................................aaaaaucUgaacgaacuuuuu.....................   8       1    seq
    ......................................................................aaaaauccAaacgaacuuuuu.....................   3       1    seq
    ......................................................................aaaaauccgaacAaacuuuuu.....................   6       1    seq
    ......................................................................aaaaauccgaacgaacuuuuug....................   443     0    seq
    ......................................................................aaaaauccAaacgaacuuuuug....................   1       1    seq
    ......................................................................aaaaauccAaacgaacuuuuugg...................   2       1    seq
    ......................................................................aaaaaucUgaacgaacuuuuugg...................   2       1    seq
    ......................................................................aaaaauccgaacgaacuuuuuggg..................   11      0    seq
    .......................................................................aaaauccgaacgaacuuuu......................   3       0    seq
    .......................................................................aaaauccgaacgaacuuuuugg...................   5       0    seq
```
